# Proposal-Free Fully Convolutional Network: Object Detection Based on a Box Map

**DOI:** 10.3390/s24113529

**Published:** 2024-05-30

**Authors:** Zhihao Su, Afzan Adam, Mohammad Faidzul Nasrudin, Anton Satria Prabuwono

**Affiliations:** 1 Center for Artificial Intelligence Technology, Faculty of Information Science and Technology, Universiti Kebangsaan Malaysia, Bangi 43600, Selangor, Malaysia; p115435@siswa.ukm.edu.my (Z.S.); mfn@ukm.edu.my (M.F.N.); 2 Faculty of Computing and Information Technology in Rabigh, King Abdulaziz University, Rabigh 21911, Saudi Arabia; aprabuwono@kau.edu.sa

**Keywords:** computer vision, object detection, deep learning algorithms, proposal-free detector

## Abstract

Region proposal-based detectors, such as Region-Convolutional Neural Networks (R-CNNs), Fast R-CNNs, Faster R-CNNs, and Region-Based Fully Convolutional Networks (R-FCNs), employ a two-stage process involving region proposal generation followed by classification. This approach is effective but computationally intensive and typically slower than proposal-free methods. Therefore, region proposal-free detectors are becoming popular to balance accuracy and speed. This paper proposes a proposal-free, fully convolutional network (PF-FCN) that outperforms other state-of-the-art, proposal-free methods. Unlike traditional region proposal-free methods, PF-FCN can generate a “box map” based on regression training techniques. This box map comprises a set of vectors, each designed to produce bounding boxes corresponding to the positions of objects in the input image. The channel and spatial contextualized sub-network are further designed to learn a “box map”. In comparison to renowned proposal-free detectors such as CornerNet, CenterNet, and You Look Only Once (YOLO), PF-FCN utilizes a fully convolutional, single-pass method. By reducing the need for fully connected layers and filtering center points, the method considerably reduces the number of trained parameters and optimizes the scalability across varying input sizes. Evaluations of benchmark datasets suggest the effectiveness of PF-FCN: the proposed model achieved an mAP of 89.6% on PASCAL VOC 2012 and 71.7% on MS COCO, which are higher than those of the baseline Fully Convolutional One-Stage Detector (FCOS) and other classical proposal-free detectors. The results prove the significance of proposal-free detectors in both practical applications and future research.

## 1. Introduction

In the realm of computer vision, object detection is one of the most important tasks, and its applications range from surveillance to autonomous driving [1]. Deep learning algorithms have achieved great success in object detection tasks, surpassing manual feature extraction algorithms [2]. With their ability to learn complex patterns from vast amounts of data, these methods have consistently outperformed traditional techniques [3,4].

Among deep learning-based object detection methods, region proposal-based detectors, such as R-CNN [5], Fast R-CNN [6], and Faster R-CNN [7], have attracted substantial interest. These methods typically operate in two stages, i.e., initial region proposal generation, followed by object classification. However, region proposal-based detectors have many problems. First, owing to their computational intensiveness, these detectors can exhibit latency, making them suboptimal for real-time applications for which swift object detection is crucial, such as autonomous driving [8,9], surveillance [10,11], motion detection [12,13], medical image detection [14,15], and traffic monitoring [16,17]. Second, because detectors based on region proposals actually make the detection problem into a multiple-classification problem, for all classification tasks, the models share the same CNN model. The CNN model classifies them independently, which causes the CNN model to process information only within the bounding boxes; examples include the Single-Shot MultiBox Detector (SSD) [18], YOLOv1 [19], R-CNN [5], the Spatial Pyramid Pooling Network (SPPNet) [6], Fast R-CNN [7], and Faster R-CNN [20]. A region proposal that is near ground truths has a high probability of being identified as an object [11,12]. However, if a region proposal includes only one object, it will also have a low probability of being identified as positive. In addition, some region proposals contain an entire object but also have other redundant background features; additionally, they can be easily identified as negatives. The regression method addressing negative predictions has not solved this problem because the limited features within the region proposal are insufficient [19]. Furthermore, postprocessing methods addressing abundant bounding boxes can reduce abundant positive predictions but not negative predictions when the ground truths are used [20]. Although researchers have attempted to solve this problem, they still cannot solve it completely [20]. Third, region proposal methods might not always be good at proposing regions for various-scale objects in an image, especially very small or large objects. This can lead to scale invariance issues, affecting detection accuracy [13].

Proposal-based detectors are dominant but not the only approach to object detection. The inefficiencies of the two-stage process gave rise to direct proposal-free detectors. By omitting the region proposal stage, these architectures, including YOLOv1 [19], CornerNet [21], CenterNet [22], FCOS [23], CentripetalNet [24], FoveaBox [25], and ObjectBox [26], offer more concise approaches, leading to reduced computation and faster processing times. However, proposal-free methods still have considerable room for innovation and optimization.

Because of the limitations presented in both proposal-based and existing proposal-free methods, this paper introduces a novel, efficient, and accurate proposal-free detection mechanism. The paper presents the Proposal-Free Fully Convolutional Network (PF-FCN), a fully convolutional network designed for object detection to balance the accuracy and speed of detectors. In addition, the paper proposes a new concept called the “bounding box filter” based on the bounding box vector, which can be utilized to reduce the number of redundant, low-quality detected bounding boxes. Because each object occupies multiple positions, PF-FCN needs to use the bounding box filter to remove redundant bounding boxes that are far from the center.

The primary contributions of this paper are as follows:

(1) This paper proposes a novel “box map” based on the channel and spatial contextualized sub-network to generate bounding boxes instead of the traditional candidate bounding box method.

(2) This paper proposes a new concept “bounding box filter” to assist in the postprocessing of redundant bounding boxes.

(3) This paper designs an end-to-end training approach to improve computational efficiency and balance accuracy and speed.

Following this introduction, Section 2 provides a detailed discussion of related work and explains the evolution of object detection algorithms. Section 3 provides an in-depth exposition of the PF-FCN architecture, its components, and its underlying principles. The experimental setups, results, and discussion are presented in Section 4 in detail. This paper summarizes the findings and offers insights into potential avenues for future research in Section 5.

## 2. Related Works

Object detection is an important research area in computer vision, given its wide range of applications, from self-driving cars [8,9] to safety systems [10,11]. The development of object detection algorithms, especially for deep learning applications, is both interesting and crucial to understanding the direction of current research; examples include EfficientDet [27], YOLOv6 [28], and YOLOv7 [29]. In this section, the paper discusses the history of deep learning detectors, focusing on two main categories, i.e., region proposal-based detectors and region proposal-free detectors.

### 2.1. Region Proposal-Based Detectors

The origins of region proposal-based detectors can be traced back to the pioneering Region-Convolutional Neural Network (R-CNN) [5] framework. R-CNN cleverly utilizes the power of CNNs for object classification, taking advantage of region proposals generated through a “selective search” method. Although pioneering, the separate steps of region proposal and classification are computationally expensive. To solve this problem, Fast R-CNN [6] was developed to simplify the process by introducing ROI pooling, a technique that can extract fixed-size features from different proposal sizes and shapes. However, because the generation of ROIs still depends on the selective search or EdgeBoxes methods, the speed of Fast R-CNN encounters a bottleneck. Faster R-CNN [20] ultimately reduces this dependence by integrating a Region Proposal Network (RPN) to predict region proposals directly from feature maps. Subsequent advances, such as Mask R-CNN [30], have extended the capabilities of Faster R-CNN by adding a segmentation mask prediction branch, allowing both object detection and instance segmentation. Furthermore, Cascade R-CNN [31] proposes a multistage framework where detection is iteratively refined. Similarly, R-FCN [13] utilizes position-sensitive score maps, thereby achieving spatial invariance.

### 2.2. Region Proposal-Free Detectors

Although the above detectors show significant improvements, the dependence on region proposals increases the computational overhead, prompting research into region proposal-free detectors. A major innovation occurred with the advent of YOLO [19,32,33]. YOLO divides an image into a grid and predicts bounding boxes using the class score of each cell to achieve unprecedented speed and accuracy. The SSD [18] extends this further by predicting multiple bounding boxes of different scales from different feature map resolutions. Recognizing the imbalance between foreground and background classes in detection tasks, RetinaNet [34] exploits focal loss, but its most significant variant, anchor-free RetinaNet, removes predefined anchor boxes, making it truly proposal-free. Notably, some detectors, such as CornerNet [21], detect objects using pairs of keypoints instead of the bounding box approach, while CenterNet [22] predicts the center of an object based on height and width, making detection a keypoint prediction problem. The FCOS [23], an anchor-free and proposal-free detector, predicts objects based on a 4D vector (l, t, r, b) encoding the size of bounding boxes, which strongly inspired the proposed PF-FCN.

The development of region proposal-free detectors reflects the pursuit of object detection efficiency. Eliminating the region proposal stage not only reduces computational complexity but also reduces potential errors based on the classification of candidates; examples include SSD [18], YOLOv1 [19], RetinaNet [34], CornerNet [21], CenterNet [22], and FCOS [23]. Furthermore, the region proposal-free approach introduces an important innovation by treating object detection as a direct-regression problem or keypoint prediction [35,36,37,38]. As real-world applications increasingly require real-time processing [39,40,41,42,43], the importance of region proposal-free detectors such as the proposed PF-FCN becomes more remarkable. In summary, from region proposal-based detectors to region proposal-free detectors, the research emphasis is always on achieving a delicate balance between accuracy and efficiency.

## 3. Method

**Overview.** In PF-FCN, a box map is designed to generate a group of vectors corresponding to each position of the input image to indicate bounding boxes. A fully convolutional network with ResNet-101 as its backbone outputs the box map. This box map is a set of tensors that are responsible for delineating the bounding boxes of detected objects. In addition to these tensors, the box map can also produce a bounding box filter map to aid in the postprocessing of abundant bounding boxes, ensuring that only the most confident detections are retained.

**Figure 1:**
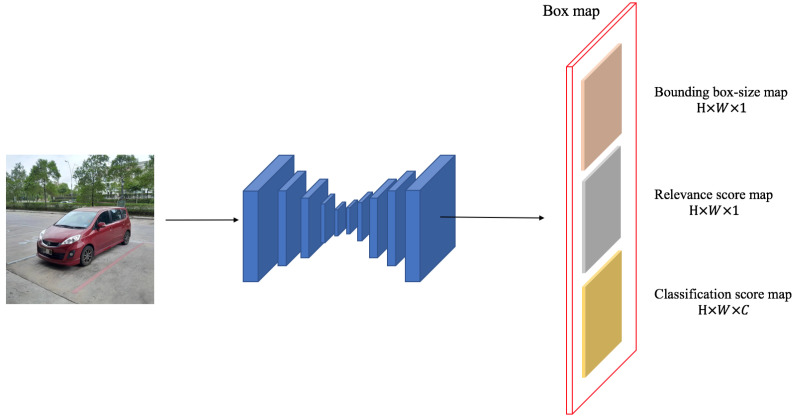
The architecture of the proposed PF-FCN.

Figure 1 provides an overview of PF-FCN. The paper employs Fully Convolutional One-Stage Detector (FCOS) as the baseline. PF-FCN utilizes ResNet-101 as a backbone to extract deep features and then generates a box map to detect objects. The box map has three outputs: a bounding box-size map, a relevance-score map, and a classification-score map. The bounding box-size map is used to generate a bounding box for detection. The relevance-score map is used to classify the foreground and background. The classification-score map predicts the category of objects. In addition, the paper proposes a “bounding box filter” to refine these detections before Non-Maximum Suppression (NMS) postprocessing. Compared with other region proposal-based detectors, PF-FCN focuses on the prediction of the box map to balance efficiency and accuracy.

**Architecture.** Usually, for region proposal-based detectors, researchers define vectors (x,y,w,h) to represent the position and size of bounding boxes. *x* represents the proportion of the horizontal position to the entire screen of the box center, *y* represents the proportion of the vertical position to the entire screen of the box center, *w* represents the width of the bounding box, and *h* represents the height of the bounding box. However, for the proposed proposal-free method, each location in the image can generate bounding boxes.

Let $F_{i}\in R^{H\times w\times C}$ be the feature maps at the layer of the network and *s* be the total stride of the convolutional operation. For each location (x,y) on the feature map, a bounding box can be generated based on the vector (l,t,r,b). If the location (x,y) is located in the ground-truth bounding boxes, the ground-truth vector $\left(\hat{l},\hat{t},\hat{r},\hat{b}\right)$ is obtained, where $\left(\hat{l},\hat{t},\hat{r},\hat{b}\right)$ represents the true distance to the left, top, right, and bottom from this position, which are distributed clockwise. The ground-truth bounding boxes for each input image are defined as $\left\{B_{i}\right\}$, where $B_{i}=\left(x^{\left(i\right)},y^{\left(i\right)},w^{\left(i\right)},h^{\left(i\right)},p{\left(c\right)}^{\left(i\right)}\right)\in R^{4}\times 1,2,\ldots ,C$. Here, $\left(x^{\left(i\right)},y^{\left(i\right)},w^{\left(i\right)},h^{\left(i\right)}\right)$ denotes the position and size of the *i*-th ground-truth bounding box. $p{\left(c\right)}^{\left(i\right)}$ is the object category in the bounding box. *C* is the number of total classes in the dataset. $R^{H\times W\times C}$ and $R^{4}$ indicate that the linear space over the real number field *R* has H×W×C and 4 dimensions, respectively.

First, to calculate the ground-truth bounding boxes $B_{i}=\left(l^{\left(i\right)},t^{\left(i\right)},r^{\left(i\right)},b^{\left(i\right)},p{\left(c\right)}^{\left(i\right)}\right)\in R^{4}\times \left\{1,2,\ldots ,C\right\}$, the paper defines the ground-truth coordinates of the left-top and bottom-right corners of the bounding box $\left(x_{0}^{\left(i\right)},y_{0}^{\left(i\right)},x_{1}^{\left(i\right)},y_{1}^{\left(i\right)}\right)$, which can be expressed as the following formula:

(1) $$\begin{array}{c} x_{0}^{\left(i\right)}=x^{\left(i\right)}-w^{\left(i\right)},y_{0}^{\left(i\right)}=y^{\left(i\right)}-h^{\left(i\right)},\\ x_{1}^{\left(i\right)}=x^{\left(i\right)}+w^{\left(i\right)},y_{1}^{\left(i\right)}=y^{\left(i\right)}+h^{\left(i\right)}. \end{array}$$

Then, based on the above vector, $\left(x_{0}^{\left(i\right)},y_{0}^{\left(i\right)},x_{1}^{\left(i\right)},y_{1}^{\left(i\right)}\right)$, if the location (x,y) is within the scope of the ground-truth bounding box, the paper can define the ground-truth bounding box vector $\left(\hat{l},\hat{t},\hat{r},\hat{b}\right)$ as Formula (2):

(2) $$\begin{array}{c} \hat{l}=x-x_{0}^{\left(i\right)},\hat{t}=y-y_{0}^{\left(i\right)},\\ \hat{r}=x_{1}^{\left(i\right)}-x,\hat{b}=y_{1}^{\left(i\right)}-y. \end{array}$$

The final box map is actually the final feature map, $F_{i}$, and the paper can map the predicted value, (l,t,r,b), back onto the input image as $\left(\left\lfloor \frac{s}{2}\right\rfloor +l\cdot s,\left\lfloor \frac{s}{2}\right\rfloor +t\cdot s,\left\lfloor \frac{s}{2}\right\rfloor +r\cdot s,\left\lfloor \frac{s}{2}\right\rfloor +b\cdot s\right)$

**Network backbone.** The baseline network is a novel proposal-free network FCOS, which provides a distinct approach to object detection. FCOS directly predicts the existence, category, and bounding box of objects at each pixel position of the grid distribution without anchor boxes, which can be regarded as a center-based detector. Compared with FCOS, the proposed PF-FCN is based on fewer center points generated via the semantic information of the object, rather than the net-like arrangement of center points.

The paper selected ResNet-101 as the backbone of PF-FCN due to its excellent depth and residual connections, which form the backbone of PF-FCN. It comprises 101 layers that are designed to extract deep and rich feature hierarchies without gradient disappearance or explosion because its fast path can retain the original features.

**Input and output.** The input is a picture. After resizing, a standard image of size 600×600 is programmed to be input into the PF-FCN model, and the output is a box map. This box map is a tensor, and the value of each tensor is a vector. This vector can generate three maps, namely, the bounding box-size map, relevance-score map, and classification-score map. This tensor is mapped to a 600×600 resized image, and the positions correspond to one to one.

**Figure 2:**
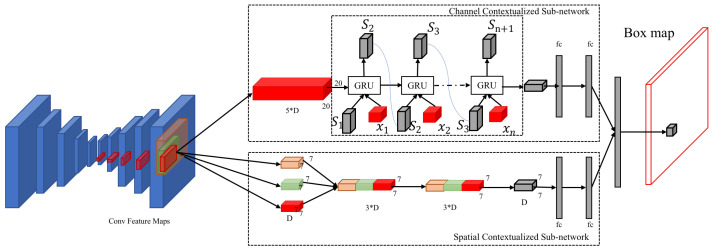
The architecture of the contextualized sub-network.

**Channel and Spatial Contextualized Sub-network.** Each value of the box map corresponds to a specific region on each feature map of FCN. We introduce a novel sub-network-comprising channel and spatial contextualization, designed to leverage the multi-level feature maps derived from the latter stages of the FCN for box map generation, as shown in Figure 2.

In Figure 2, different color of feature maps means different positions corresponding to the input image, and the arrow means changing the size of the feature map through upsampling or downsampling. The letter “D” represents the value of one dimension of the extracted feature map, specifically the number of channels. “$S_{n}$” denotes the value of the state layer in the recurrent neural network (RNN). This state layer value, combined with the input layers of the next iteration, is fed into the RNN. We use the Gated Recurrent Unit (GRU) as the cell due to several advantages that it offers. GRUs are known for their ability to capture dependencies in sequential data effectively while maintaining a simpler architecture compared to Long Short-Term Memory (LSTM) units [39]. This simplicity translates into fewer parameters, making GRUs computationally more efficient, which is particularly beneficial in scenarios with limited computational resources. Additionally, GRUs often converge faster and require less training time, making them an attractive choice for our application. Regular ResNet or DenseNet is used for feature extraction, but we employed FCN for this purpose. In the latter part, the feature maps are of different sizes. We aim to leverage the contextual relationships of different feature maps, which necessitates the use of GRU for modeling. This allows us to capture the relationship between each region and its surrounding areas, as well as the feature maps before and after convolution operations. GRU offers several advantages over LSTM, including a simpler architecture with fewer parameters, which leads to faster convergence and lower computational requirements, making it more efficient for our needs [40].

This sub-network has two principal components: a channel-contextualization sub-network and a spatial-contextualization sub-network. The channel-contextualization sub-network is engaged to extract inter-channel contextual features. To facilitate this, we utilize an upsampling strategy to normalize the spatial dimensions of feature maps across five distinct levels. These uniformly sized feature maps are then sequentially fed into a GRU layer to assimilate contextual information for each channel effectively. Conversely, the spatial contextualization sub-network is tailored to extrapolate spatial contextual features from the final layer of the feature maps. It amplifies the receptive field incrementally—doubling and then tripling—to encompass broader contextual features. Following this expansion, a downsampling technique is applied to amalgamate the three feature maps. A convolution operation is subsequently exploited to distill common features across these regions, thereby enriching the extraction of spatial contextual details at targeted locations. Finally, the outputs of both contextual sub-networks are amalgamated to determine the values associated with the box map. This integrated approach ensures that our model comprehensively captures and utilizes contextual information from the feature maps to accurately generate the box map.

**Box map.** The core innovation of this paper is the box map, which has three outputs, i.e., the bounding box-size map, the relevance-score map, and the classification-score map. The box map is similar to the position-sensitive score map in R-FCN. The position-sensitive score map of R-FCN classifies positions into only the foreground and the background, while PF-FCN classifies positions into categories of objects and generates bounding boxes [13]. Since the distribution of all the values on the box map corresponds to the position of the input image, each of the three output value distributions is responsible for the position of the corresponding input image. The paper sets the box map output to be a k×k matrix, and the value of each matrix is $Y_{i,j}\left(0\leq i,j\leq k-1\right)$, and then it defines $\left(l_{i,j},t_{i,j},r_{i,j},b_{i,j}\right)$ as the bounding box-size map, $R_{i,j}$ as the relevance-score map, and $p{\left(c\right)}_{i,j}$ as the classification-score map. The formula of the box maps can be expressed as Formula (3).

(3) $$\begin{array}{c} Y_{i,j}=\left(l_{i,j},t_{i,j},r_{i,j},b_{i,j},R_{i,j},p{\left(c\right)}_{i,j}\right)\in R^{5}\times \left\{1,2,\ldots ,C\right\} \end{array}$$

The bounding box-size map is the core of the box map. Each value, $\left(l_{i,j},t_{i,j},r_{i,j},b_{i,j}\right)$, is a vector, and (l,t,r,b) indicates the relative position and size of the bounding box that needs to be generated, where (l,t,r,b) represents the distance to the left, top, right, and bottom from this position, which are distributed clockwise. This vector can produce a bounding box. FCOS generates bounding boxes directly on all the pixels of the feature map [23], resulting in a large number of calculations, while the bounding box-size map is scalable to generate fewer bounding boxes.

**Figure 3:**
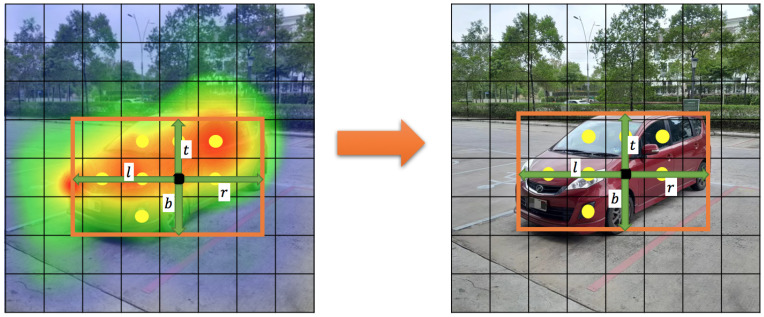
Heatmap visualization of the relevance-score map.

The values in the relevance score map, $R_{i,j}$, are all 0–1, indicating how relevant the corresponding position is to the object. If the grid cell is near the center of the object, the likelihood of generating a bounding box is high. If the grid cell is near the edge of the object, the likelihood of generating a bounding box is low. The value of $R_{i,j}$ increases linearly from 0 to 1 from the edge box of the object to the center box. Then, divide the picture into grid cell areas, perform max pooling on the *R* value within the area, and take the maximum value as the *R* value of the cell grid, and set a threshold. Additionally, the paper defines a new concept, the “R-threshold”, which means that, if the *R* value of the grid cell exceeds the R-threshold, the center point will be retained, and bounding boxes will be generated, which is shown in Figure 3. The green arrows in Figure 3 mean the value of (l,t,r,b).

Each value of the classification-score map $p{\left(c\right)}_{i,j}\in \left\{1,2,\ldots ,C\right\}$ is a vector. This vector is the value of the confidence of all categories. The class corresponding to the highest default value in the vector indicates the category of the corresponding box of this box map.

**Loss function.** The output $Y_{i,j}$ consists of k×k bins, and each bin has one value, which is $\left(l_{i,j},t_{i,j},r_{i,j},b_{i,j},R_{i,j},p{\left(c\right)}_{i,j}\right)$. $\left(l_{i,j},t_{i,j},r_{i,j},b_{i,j}\right)$ represents the size of the bounding boxes. $p{\left(c\right)}_{i,j}$ represents the probability of each object. The indice (i,j) in $Y_{i,j}$ represents the position of the bin. *R* is the probability that the position is relevant to the object. The paper defines the loss value of PF-FCN in the training stage as *L*, so the loss function is defined as Formula (5).

(4) $$\begin{array}{c} L=\lambda_{coord}\sum_{i=0}^{k}\sum_{j=0}^{k}\alpha_{i,j}^{obj}[{\left(t_{i,j}-{\hat{t}}_{i,j}\right)}^{2}\\ +{\left(b_{i,j}-{\hat{b}}_{i,j}\right)}^{2}+{\left(l_{i,j}-{\hat{l}}_{i,j}\right)}^{2}+{\left(r_{i,j}-{\hat{r}}_{i,j}\right)}^{2}]\\ +\sum_{i=0}^{k}\sum_{j=0}^{k}\alpha_{ij}^{obj}{\left(R_{i,j}-{\hat{R}}_{i,j}\right)}^{2}\\ +\lambda_{\mathrm{noobj}\;}\sum_{i=0}^{k}\sum_{j=0}^{k}\alpha_{ij}^{\mathrm{noobj}\;}{\left(R_{i,j}-{\hat{R}}_{i,j}\right)}^{2}\\ +\sum_{i=0}^{k}\sum_{j=0}^{k}\alpha_{ij}^{obj}\sum_{c\in \;\mathrm{class}\;}{\left(p{\left(c\right)}_{i,j}-\hat{p}{\left(c\right)}_{i,j}\right)}^{2} \end{array}$$

If the grid cell is responsible for this object, $\alpha_{ij}^{obj}$ is 1; otherwise, it is 0. If the grid cell is not responsible for this object, $\alpha_{ij}^{noobj}$ is 1; otherwise, it is 0. Additionally, $\lambda_{coord}$ is the weight of the localization loss. Then, set $\lambda_{coord}$ to 5 to give a greater weight to the position regression, and set $\lambda_{noobj}$ to 0.5 to ignore unimportant grid cells.

**Figure 4:**
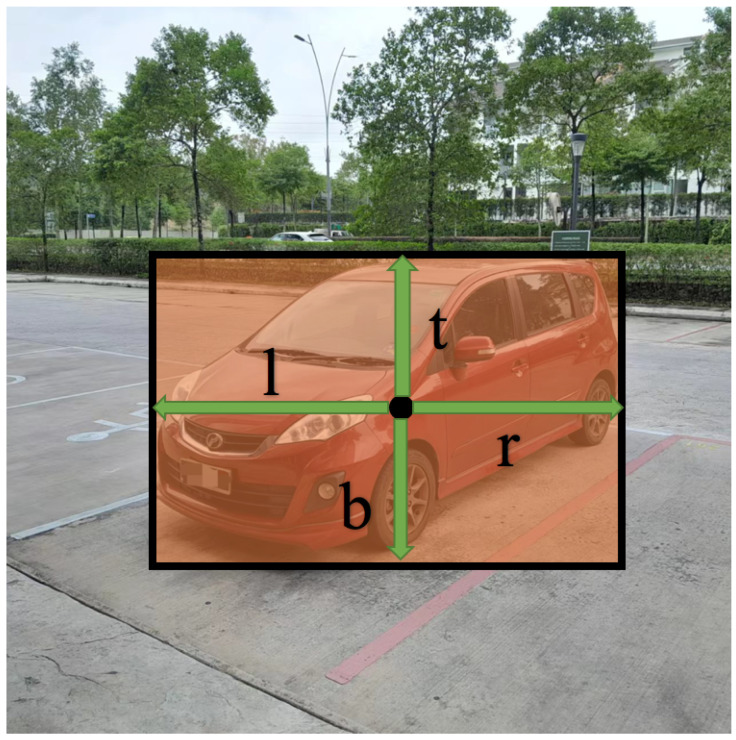
Calculation method for the bounding box filter.

**Bounding box filter**. The proposed object detection algorithm produces a large number of redundant candidate-bounding boxes near the object. Therefore, a new concept, the “bounding box filter”, is proposed to reduce the number of redundant, low-quality detected bounding boxes, which is shown in Figure 4. The green arrows in Figure 3 mean the value of (l,t,r,b). The design was inspired by the center-ness of FCOS, but the proposed bounding box filter has simpler calculations and saves computing resources. Since each relevant position generates a bounding box, PF-FCN can use the bounding box filter to remove these redundant bounding boxes. The score of the bounding box filter is defined as Formula (6).

(5) $$\begin{array}{c} \theta =16*\left(\frac{l}{l+r}\times \frac{r}{l+r}\times \frac{t}{t+b}\times \frac{b}{t+b}\right),\theta \in \left[0,1\right] \end{array}$$

The normalized coefficient 16 makes θ more meaningful, and its value range is controlled between 0 and 1. The minimum value is 0, and the maximum value is 1. If the position is at the center of the object, the value is 1; if the position is on the edge of the object, the value is 0. A threshold can be given to remove some low-quality detected bounding boxes.

According to the formula of the bounding box filter, the initial values range between 0 and 1/16, with the maximum value being 1/16 when the detection point is precisely at the center. To make these values more intuitive and within a 0 to 1 range, we multiply them by 16. If an object is very small and falls near a corner, the grid at the corner will take responsibility, positioning the center point at the middle of the grid and generating the (l,t,r,b) values accordingly. These (l,t,r,b) values will also be proportionally smaller. Conversely, if the object is large and falls near a corner, the responsibility will shift to the grid closer to the center of the object. This approach aims to produce (l,t,r,b) values that generate bounding boxes that are as balanced as possible, reflecting the true size and position of the object.

This value does not require training because it is determined only according to (l,t,r,b). Finally, the score of the bounding box filter is calculated from the predicted (l,t,r,b) values, which can help remove some inferior detected bounding boxes.

**Figure 5:**
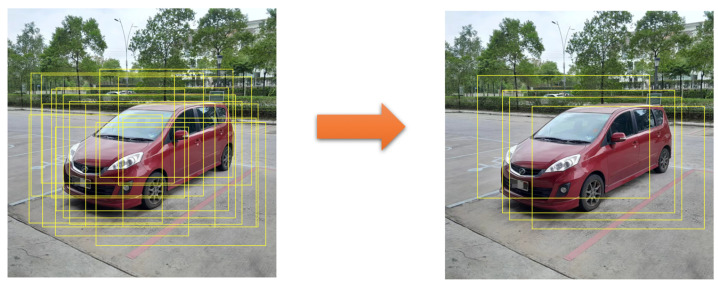
Candidate bounding boxes before (**left**) and after (**right**) the filtering of the bounding box filter.

Figure 5 shows the postprocessing of bounding boxes based on the bounding box filter. First, lots of bounding boxes are generated for each object. However, the bounding boxes generated at the positions near the edge of objects are redundant. Then, PF-FCN eliminates redundant boxes by setting the threshold of the bounding box filter. The orange in Figure 5 arrows mean the processing of the bounding box filter.

The bounding box filter performs an initial assessment by determining whether the generated box’s position is reasonable without needing to compare it with other boxes. It deletes bounding boxes that are generated in edge regions away from the ground truths. In contrast, Non-Maximum Suppression (NMS) requires comparing two different boxes to each other. After the bounding box filter has made its preliminary selections, NMS is then used to perform the final selection process.

**Proposal-free regression mechanism.** The proposed approach differs from proposal-based detectors in that the regression mechanism directly maps image pixels to the bounding box coordinates and confidence scores of the box map. This is achieved through the architecture of the PF-FCN network and the design of its final layers, which produce the output tensor of shape k×k. Each value is an $\left(l_{i,j},t_{i,j},r_{i,j},b_{i,j},R_{i,j},p{\left(c\right)}_{i,j}\right)$ vector. Regression tasks are essentially trained to minimize the difference between these predicted and ground-truth values during training.

**Figure 6:**
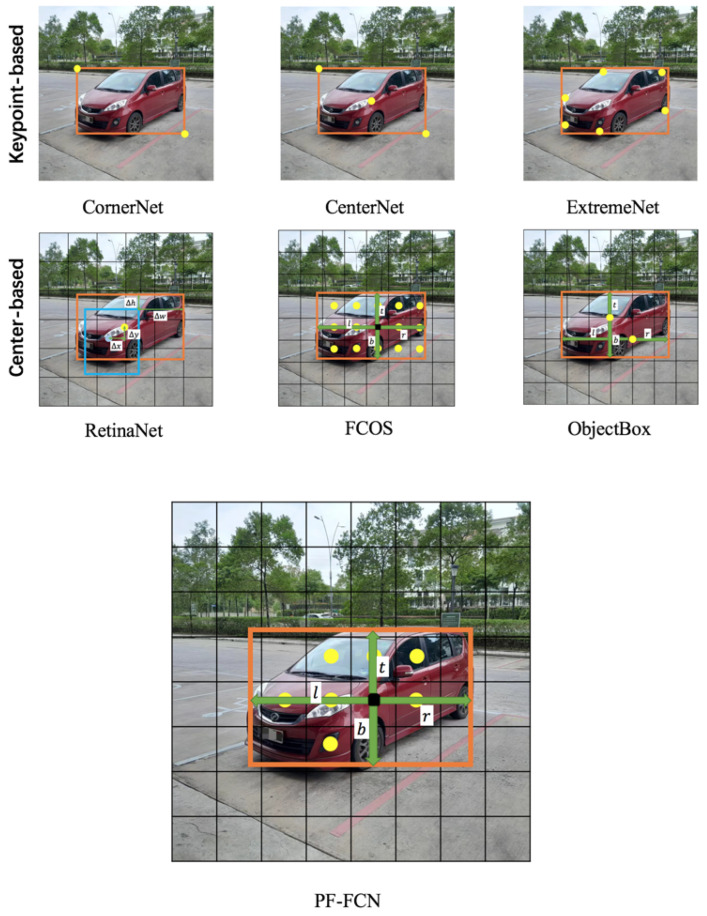
The comparison of bounding box generation methods among different deep-learning detectors.

**Comparison with other proposal-free detectors.** CornerNet, CenterNet, ExtremeNet, RetinaNet, FCOS, and ObjectBox are classic proposal-free detectors shown in Figure 6, each of which adopts unique key technologies to improve detection accuracy and efficiency. These algorithms can be divided into keypoint-based methods and center-based methods whose differences are ways to locate and identify objects in images.

Detectors based on key points include CornerNet, CenterNet, and ExtremeNet. CornerNet localizes objects by detecting two corner points of the bounding box, i.e., the upper left corner and the lower right corner, which does not use anchor boxes and reduces the complexity of the prediction process. CenterNet locates objects by directly predicting the location of the center point of an object, as well as the size of the object, simplifying the detection process and improving speed and accuracy. ExtremeNet detects objects based on their center points and the four extreme points, including the top, bottom, left, and right, which is especially effective for complex-shaped object detection.

Center-based algorithms include RetinaNet, FCOS, and ObjectBox. RetinaNet uses feature pyramids and focal loss to solve the category-imbalance problem and improve the detection performance of small objects. Although it is not a complete center point-based method, efficient detection results are obtained by optimizing the detection process. FCOS is a fully convolutional, single-stage object detection algorithm that does not use anchor boxes at all, and it directly predicts the bounding box and category of an object at each pixel position to improve the flexibility and efficiency of detection. ObjectBox adopts an anchor-free, single-stage object detection method, which processes all objects at different scales equally, using the object center position as a shape- and size-insensitive anchor point for label assignment. Furthermore, it introduces a new regression objective and a customized IoU loss to effectively handle scale-changing objects, avoiding dataset-dependent hyperparameter adjustments.

The proposed PF-FCN engages a novel box map to generate the center point and the size of its bounding box in the center area of the object, based on the semantic information of objects, and it proposes a bounding box filter that saves a lot of computing resources. Overall, different algorithms demonstrate the diverse approaches in the field of object detection from keypoint-based to center-based. Keypoint-based algorithms locate objects by identifying specific image feature points, while center-based algorithms focus on optimizing the entire detection process to improve detection efficiency and accuracy. The selection and application of each algorithm depend on the requirements of the specific task and the constraints of the actual application scenario.

**Advantages.** The proposed PF-FCN method uses an FCN as the overall framework and designs the box map as the output. The proposed method has many advantages, such as end-to-end training and feature spatial invariance. PF-FCN is designed to process the entire image in a single forward propagation without any sliding-window operations, which allows for end-to-end training and prediction, making the process computationally efficient. In addition, for region proposal-based detectors, most of the computations occur in fully connected layers, which are applied independently to each region proposal. Through the FCN backbone, PF-FCN can distribute computations more evenly across the spatial dimensions of the input. In summary, the box map provides an effective and computationally efficient detection method, and the structure of the FCN provides the advantage of feature spatial invariance.

## 4. Experiments

### 4.1. Datasets

MS COCO [44] is a large dataset used for a variety of computer vision tasks, including object detection, segmentation, and captioning. It is designed to evaluate state-of-the-art algorithms in more diverse and complex scenarios, such as object detection, semantic segmentation, instance segmentation, keypoint detection, content segmentation, and image captioning. This dataset contains more than 200,000 labeled images with more than 1.5 million object instances distributed across 80 object categories.

The PASCAL Visual Object Class (VOC) 2007 [45] dataset is one of the most popular datasets in the VOC series; it contains 9963 images, including more than 24,000 object instances in 20 categories, for object classification and detection. PASCAL VOC 2012 is a follow-up version of the PASCAL VOC challenge based on the 2007 dataset. VOC 2012 contains 11,530 images with more than 27,450 object instances in the same 20 categories as VOC 2007 for object classification, detection, and segmentation. Like in VOC 2007, each image in VOC 2012 can belong to multiple classes, and annotations are provided for class labels and bounding boxes. Additionally, VOC 2012 provides segmentation masks for objects.

### 4.2. Evaluation Metrics

The intersection over union (IoU) [1] measures the overlap between two bounding boxes (e.g., ground truth and predicted boxes), and it is defined as Formula (7); the IoU ranges from 0 (no overlap) to 1 (complete overlap).

(6) $$\begin{array}{c} IoU=\frac{groundtruth\cap predictedbox}{groundtruth\cup predictedbox} \end{array}$$

The mean average precision (mAP) is a widely used metric in object detection that summarizes the average precision (AP) across all categories. For each class, the area under the precision–recall curve is calculated, which yields the AP for that class. The mAP is the average AP across all categories. The higher the mAP, the better the overall detection performance in all the categories. Generally, AP is in a single category, and mAP is the average AP value in all categories. However, in the context of other related papers, AP is the mAP. For example, in [13,21], the average AP in all the categories was calculated, and the AP in these studies was considered the mAP.

$AP_{0.5}$ indicates the mAP when the IoU threshold is 0.5. This approach has been standard for many years, especially for the PASCAL VOC dataset. The $mAP_{s}$, $mAP_{m}$, and $mAP_{l}$ metrics provide mAP values for different object sizes: $mAP_{s}$, small objects; $mAP_{m}$, medium objects; and $mAP_{l}$, large objects. These methods realize detailed performance for objects of different sizes.

$AP_{0.5∼0.95}$ are average mAPs calculated at various IoU thresholds between 0.5 and 0.95 at intervals of 0.05. This approach provides a more comprehensive view of the model’s performance at different bounding box overlap levels.

Frames per second (FPS) [19] indicate the number of images (frames) that the model can process in one second. A higher FPS indicates faster processing, which is crucial for real-time applications.

### 4.3. Experiments on MS COCO

Models were trained on the 80 k sample training set and evaluated using the 40 k sample validation set and 20 k sample test set.

**Performance overview.** Table 1 shows that PF-FCN achieved 53.6% accuracy $AP_{0.5∼0.95}$, exceeding the baseline FCOS, which lagged behind 44.7%. The bold data representation in the table means the best result in the same metrics. According to Table 1, the overall average accuracy (AP) metric, especially $AP_{0.5∼0.95}$, is a key benchmark for evaluating the effectiveness of object detectors under different IoUs. In this case, PF-FCN achieved a convincing performance of 53.6%, which greatly exceeded that of the baseline FCOS (44.7%). According to $AP_{0.5}$, the performance of PF-FCN was 71.7%, which again proved its strength, while the performance of FCOS was 64.1%. Although PF-FCN algorithm achieves an improvement in detection speed of 26 frames/second, it lags behind the other AP metrics.

The experimental results in Table 1 reveal significant differences in the proposal-free method. The proposed PF-FCN performs best at all metrics, $\left(AP_{0.5∼0.95},AP_{0.5},AP_{s},AP_{m},AP_{l}\right)$, and only lags behind YOLOv2 and YOLOv7 in its frame rate (FPS), indicating excellent overall performance in terms of accuracy and speed. FCOS, CornerNet, and CenterNet show moderate performance in accuracy, but they lag significantly behind YOLOv7 and PF-FCN. These methods also show a lower FPS than YOLOv7. FoveaBox shows balanced performance in different object-size accuracy.

The comparison using the MS COCO dataset highlights several standout results in terms of accuracy and speed among various deep learning-detection algorithms. Notably, PF-FCN achieves the best performance across multiple metrics, with an $AP_{0.5∼0.95}$ of 53.6%, an $AP_{0.5}$ of 71.7%, and an $AP_{s}$ of 37.6%. This indicates that PF-FCN is highly effective in detecting objects of various sizes and maintains high precision at different intersection over union thresholds. YOLOv8 and YOLOv9 also demonstrate impressive results, with $AP_{0.5∼0.95}$ scores of 52.9% and 53.0%, respectively, and $AP_{0.5}$ scores of 69.8% and 70.2%, showing their robustness and accuracy. YOLOv8 further excels in detecting medium and large objects with an $AP_{m}$ of 59.3% and an $AP_{l}$ of 70.7%, the highest among all listed methods. Moreover, YOLOv7 stands out for its remarkable speed, achieving 160 FPS, making it the fastest algorithm in the comparison.

**Figure 7:**
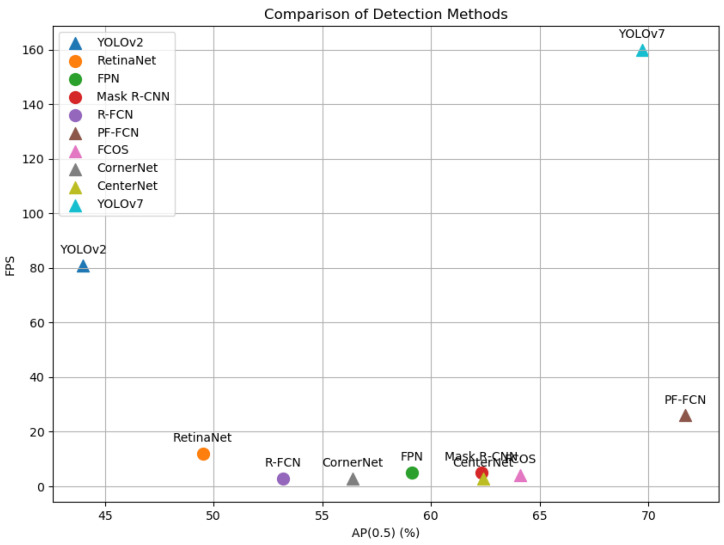
Balance of accuracy and speed on the MS COCO dataset (the triangles represent proposal-free detectors, and the circles represent proposal-based proposals).

Figure 7 visualizes Table 1 by setting the accuracy indicator $AP_{0.5}$ as the horizontal ordinate and the speed indicator FPS as the vertical ordinate, which indicates the balance of accuracy and speed on the MS COCO dataset. PF-FCN visually outperforms FCOS and CenterNet in terms of both accuracy and speed. In addition, PF-FCN also outperforms YOLOv7 in accuracy, while YOLOv7 tops the list in detection speed.

**Analysis.** An important advantage of PF-FCN is its ability to balance accuracy and speed. Across all the evaluation metrics, PF-FCN always has advantages over FCOS. This advantage is further emphasized by an $AP_{0.5}$ score of 71.7%, compared to 64.1% for FCOS. When detecting objects of different sizes, the performance of PF-FCN in the $AP_{s}$ and $AP_{m}$ metrics reaffirms its competitive advantage, especially for small and medium-sized objects, indicating that its design is robust and comprehensive. Furthermore, because of its region proposal-free nature, PF-FCN retains the advantage of computational efficiency and simplifies the detection process by combining the proposal step and the classification step based on the regression method. The real advantage of PF-FCN is the excellent balance between accuracy and speed. Although PF-FCN is not proposal-based, it competes with many proposal-based methods in terms of accuracy, such as R-FCN and FPN [38]. Proposal-based methods such as Mask R-CNN still have an advantage in terms of the AP metric. Additionally, PF-FCN achieves a faster speed of 26 FPS than the baseline R-FCN and other classical proposal-based methods. In addition, among proposal-free detectors, PF-FCN surpasses FCOS, CornerNet, CenterNet, and FoveaBox in both accuracy and speed. Too many center points generated via FCOS will lead to a serious imbalance between positive and negative samples. The corner points detected via CornerNet need to go through a complex pairing mechanism to form the final bounding box, which increases the complexity of post-processing. CenterNet may be affected by occlusion and object density in some complex scenes. FoveaBox predicts category and bounding box positions on the feature map directly, so it may encounter feature alignment problems. Small deviations between the predicted position and the actual position may affect the final detection accuracy. The proposed PF-FCN overcomes the problems of positive and negative sample imbalance and inaccurate key point positioning in complex scenes through the box map.

However, despite the above advantages, PF-FCN still faces some challenges. While it successfully outperforms many state-of-the-art methods, it does not dominate across all the metrics. The leadership of YOLOv7 in accuracy and speed is due to several trainable bag-of-freebies methods. Because PF-FCN consumes a large amount of computing resources when producing the relevance score map, the speed lags far behind YOLOv7.

### 4.4. Experiments on the PASCAL VOC Dataset

This study utilized the PASCAL VOC dataset to train and test the models. The study trained models on the union set of the VOC 2007 trainval and VOC 2012 trainval (“07 + 12”) datasets and evaluated them using the union set of the 07 trainval+test and 12 trainval datasets. The “+” of “07 + 12 + COCO” means that the training dataset is the combined dataset, including PASCAL VOC 2007 trainval and 2012 trainval, and MS COCO training set.

**Performance overview.** As Table 2 shows, the baseline model FCOS, another proposal-free method, has an $AP_{0.5}$ of 87.3%, which is slightly higher than Faster R-CNN. The bold data representation in the table means the best result in the same metrics. The average precision (AP), with a threshold of 0.5, is a key benchmark in the field of object detection, and the proposal-free model PF-FCN delivers an impressive $AP_{0.5}$ of 89.6%, surpassing FCOS. PF-FCN also has the highest frames per second (FPS) of 25. So, PF-FCN outperforms all other methods in both accuracy and speed, demonstrating significant improvements in detection performance. Among the proposal-based methods, Faster R-CNN leads with an 83.8% AP, but its FPS is extremely low at 0.3. Although Faster R-CNN is relatively accurate, it is not suitable for scenarios that require real-time processing. R-FCN lags behind Faster R-CNN in terms of AP at 82%, but its FPS is significantly higher at 5.88, which is still worse than the proposal-free approach.

On the PASCAL VOC dataset, the proposal-free methods generally outperform proposal-based methods in terms of accuracy. PF-FCN again emerges as the top performer with an $AP_{0.5}$ of 89.6%, highlighting its superior detection capabilities. CenterNet and FCOS also perform very well, with $AP_{0.5}$ scores of 87.1% and 87.3%, respectively. These results underline the effectiveness of these algorithms in providing high precision. While SSD is the fastest with 46 FPS, it lags in accuracy compared to PF-FCN, CenterNet, and FCOS. YOLOv1 and SSD are notable for their speed, processing images at 45 and 46 FPS, respectively, though their accuracy, with $AP_{0.5}$ scores of 63.4% and 74.3%, is lower than that of the top performers.

**Figure 8:**
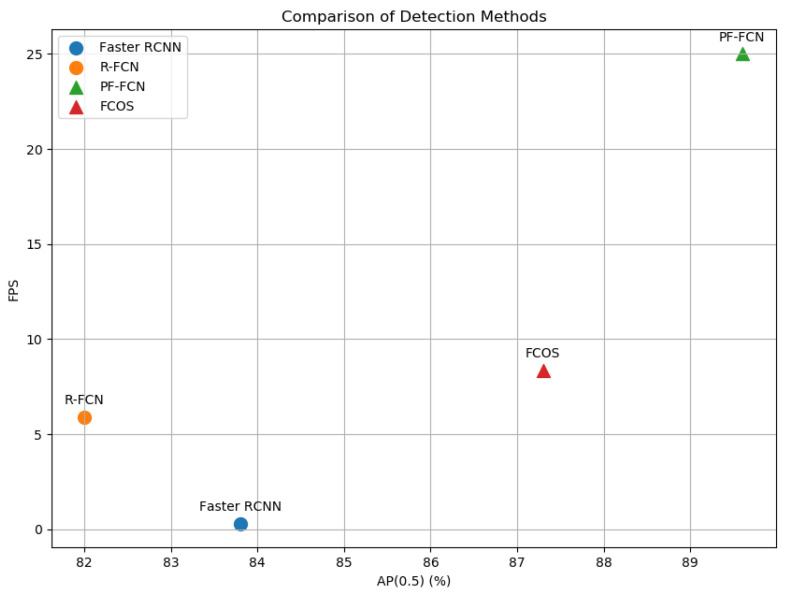
Balance of accuracy and speed on the PASCAL VOC dataset (the triangle represents a proposal-free detector, and the circles represent proposal-based detectors).

Figure 8 visualizes Table 2 by setting the accuracy indicator $AP_{0.5}$ as the horizontal ordinate and the speed indicator FPS as the vertical ordinate, which indicates the balance of accuracy and speed using the PASCAL VOC dataset. PF-FCN visually outperforms FCOS and other proposal-based methods in terms of both accuracy and speed.

**Analysis.** Although accuracy is critical, the test time efficiency per image cannot be ignored, especially in real-world applications that require real-time detection. The real advantage of PF-FCN is its efficiency. The test time per photo is only 0.04 s, far exceeding that of its peers. This superior speed, coupled with commendable accuracy, highlights the power of the proposal-free mechanism. Although Faster R-CNN has the highest accuracy, 3.36 s are required per image, which may be impractical for real-time scene detection. FCOS achieves a significant improvement in speed and only takes 0.12 s to process an image. On the other hand, although $AP_{0.5}$ performs well, there is still a slight difference between PF-FCN and Faster R-CNN models. This demonstrates the potential scope for improving model accuracy.

Among deep-learning object detectors, PF-FCN is a balanced solution that links the efficiency of proposal-free methods with the accuracy of proposal-based methods. PF-FCN outperforms the baseline FCOS and achieves competitive results against other advanced networks. The superior performance of PF-FCN may be attributed to efficient feature extraction and object localization techniques without region proposals. By eliminating the proposal generation and proposing center-point selection steps, PF-FCN can filter center points and directly predict object categories and locations to improve the AP and FPS. FCOS demonstrates the advantages of the proposal-free approach due to its highest accuracy and FPS.

Proposal-based detectors achieve lower accuracy because the proposal generation stage produces too many negatives near ground truths. Faster R-CNN has an extremely low FPS because its region proposal network (RPN) has expensive computation. R-FCN achieves a balance between accuracy and speed in a proposal-based approach. It utilizes location-sensitive score maps in region proposals, which can improve localization accuracy. However, R-FCN still involves region proposals, which inherently limits its speed compared to proposal-free methods.

Overall, proposal-free methods, especially PF-FCN, achieve higher accuracy and faster processing than proposal-based methods because their simplified architecture reduces computational requirements when maintaining or even improving detection performance.

### 4.5. Overall Performance with the MS COCO and PASCAL VOC Dataset

**Figure 9:**
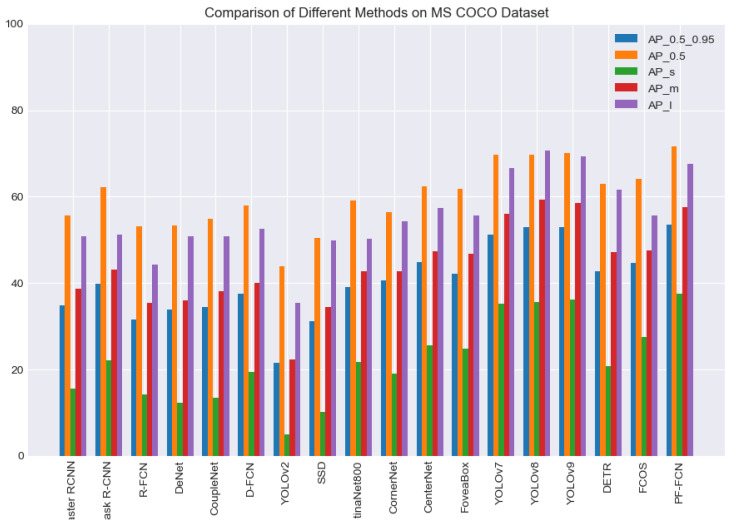
Performance visualization with the MS COCO dataset.

The comparison using the MS COCO dataset shows significant differences in accuracy and speed among various deep-learning detection algorithms, which is shown in Figure 9. Faster R-CNN and Mask R-CNN, using ResNet-101 and ResNeXt-101 backbones, respectively, achieved a 34.9% and 39.8% $AP_{0.5∼0.95}$, and a 55.7% and 62.3% $AP_{0.5}$, but their FPS was only 5, which is shown in Table 1. R-FCN performed relatively poorly, with a 31.5% $AP_{0.5∼0.95}$, a 53.2% $AP_{0.5}$, and an FPS of around 3. Among the proposal-free methods, the latest versions of the YOLO series (YOLOv7, YOLOv8, and YOLOv9) stand out in both accuracy and speed, particularly YOLOv8 and YOLOv9, which achieved a 52.9% and 53.0% $AP_{0.5∼0.95}$, respectively, with an $AP_{0.5}$ over 70%. Additionally, YOLOv7 has a significant speed advantage, reaching 160 FPS. However, methods like CenterNet and FCOS, though performing well in accuracy with an $AP_{0.5}$ of 62.4% and 64.1%, respectively, are slower. Overall, PF-FCN performs best in terms of accuracy, with an $AP_{0.5∼0.95}$ of 53.6% and an $AP_{0.5}$ of 71.7%, and it shows excellent performance across different object sizes.

**Figure 10:**
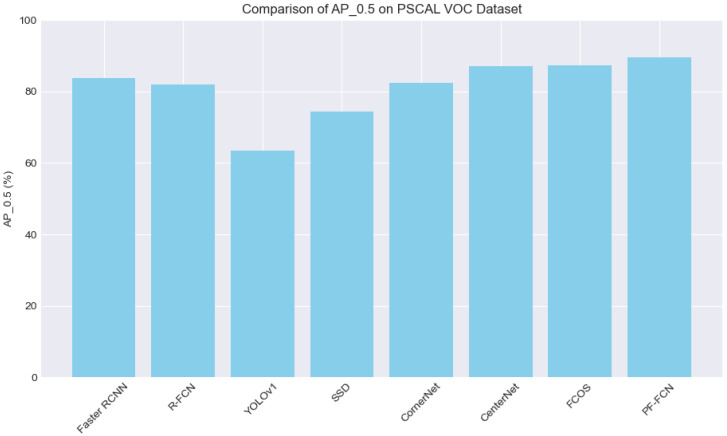
Performance visualization with the PASCAL VOC dataset.

With the PASCAL VOC dataset, proposal-based methods Faster R-CNN and R-FCN achieved an $AP_{0.5}$ of 83.8% and 82.0% respectively, which is shown in Figure 10. But their speeds are slow, with each image taking 3.36 s and 0.17 s, respectively, which is shown in Table 2. Among proposal-free methods, YOLOv1 and SSD have significant speed advantages, reaching 45 and 46 FPS, respectively, but their accuracy is lower, with an $AP_{0.5}$ of 63.4% and 74.3%. In contrast, CenterNet and FCOS performed better in accuracy, with an $AP_{0.5}$ of 87.1% and 87.3%, respectively, and their speeds were moderate, as they processed each image in 0.14 s and 0.12 s, respectively. PF-FCN stands out for this dataset, achieving an $AP_{0.5}$ of 89.6% and also being faster, processing each image in only 0.04 s, reaching 25 FPS. Overall, PF-FCN offers the best combination of high accuracy and fast speed with the PASCAL VOC dataset.

From the comparison of these two figures, it is clear that different algorithms have their own strengths and weaknesses with different datasets. Choosing the right algorithm requires considering both accuracy and speed, as well as the specific application scenario and requirements.

#### 4.5.1. Comparison of Different FCN-Based Deep-Learning Detectors

FCOS is the baseline of PF-FCN, which is one of many famous FCN-based deep-learning detectors, including R-FCN, SSD, etc. Based on the two tables provided, our PF-FCN demonstrates clear advantages in both accuracy and speed compared to other FCN-based deep learning detectors.

With the MS COCO dataset, PF-FCN achieves an $AP_{0.5∼0.95}$ of 53.6%, which is the highest among all listed methods. Additionally, it attains an $AP_{0.5}$ of 71.7%, an $AP_{s}$ of 37.6%, an $AP_{m}$ of 57.5%, and an $AP_{l}$ of 67.7%, indicating superior performance across small, medium, and large objects. Furthermore, PF-FCN operates at 26 FPS, making it one of the faster models, though not the fastest. Comparatively, while YOLOv7 achieves an impressive 160 FPS and YOLOv8 has a high accuracy with an $AP_{0.5}$ of 69.8%, neither matches the balanced performance of PF-FCN across all accuracy metrics.

With the PASCAL VOC dataset, PF-FCN again excels with an $AP_{0.5}$ of 89.6%, which is the highest among the methods compared. The time per image for PF-FCN is 0.04 s, resulting in 25 FPS, indicating a strong balance of high accuracy and reasonable speed. In comparison, SSD achieves a 46 FPS but has a significantly lower $AP_{0.5}$ of 74.3%. CenterNet and FCOS, with $AP_{0.5}$ scores of 87.1% and 87.3% respectively, also fall short of PF-FCN’s accuracy despite their competitive speeds.

These results illustrate that PF-FCN not only outperforms other FCN-based detectors in terms of precision but also maintains a competitive speed, making it highly effective for object-detection tasks. This combination of high accuracy and adequate speed underlines the robustness and efficiency of PF-FCN in various applications.

#### 4.5.2. Performance in New Specific Dataset

This paper proposes a proposal-free, fully convolutional network (PF-FCN), which outperforms other state-of-the-art proposal-free methods. Unlike traditional region proposal-free methods, PF-FCN can generate a “box map” based on regression training techniques. This box map comprises a set of vectors, each designed to produce bounding boxes corresponding to the positions of objects in the input image. The channel and spatial contextualized sub-network are further designed to learn the “box map”. PF-FCN’s unique approach makes it particularly advantageous in specific applications such as face detection, vehicle detection, and fire monitoring. In face detection, PF-FCN’s ability to accurately detect small and intricate features ensures reliable performance even in crowded or poorly lit environments. For vehicle detection, PF-FCN’s proficiency in identifying objects of various sizes and shapes supports real-time traffic monitoring and autonomous driving systems. Additionally, in fire monitoring, PF-FCN’s robust detection capabilities enable the precise identification of fire and smoke, facilitating early warning systems and improving response times to potential hazards. These specialized applications underscore PF-FCN’s versatility and superior performance across a range of object detection tasks.

#### 4.5.3. Performance in Rare Object Detection

Table 1 demonstrates the superior performance of PF-FCN (Proposal-Free Fully Convolutional Network) in detecting rare or small objects, which are often the most challenging to identify. Specifically, in the context of the MS COCO dataset, $AP_{s}$ (average precision for small objects) is a crucial metric for evaluating performance using these rare and small-scale objects.

PF-FCN achieves an impressive $AP_{s}$ score of 37.6%, which is the highest among all methods listed. This indicates that PF-FCN excels in detecting small objects, outperforming other state-of-the-art methods. For instance, YOLOv8 and YOLOv9, which are also strong performers, achieve $AP_{s}$ scores of 35.7% and 36.2%, respectively. Despite their competitive accuracy in general object detection ($AP_{0.5∼0.95}$ of 52.9% and 53.0%), they do not surpass PF-FCN’s ability to accurately detect small objects.

Comparatively, other methods, such as CenterNet and FCOS, achieve $AP_{s}$ scores of 25.6% and 27.6%, respectively, indicating a significant gap in performance for small-object detection. Even high-speed models like YOLOv7, which achieves 160 FPS, only manage an $AP_{s}$ of 35.2%, highlighting that, while speed is a strong aspect, it does not necessarily translate to better accuracy for small objects.

The exceptional performance of PF-FCN in $AP_{s}$ can be attributed to its unique approach. PF-FCN generates a “box map” through regression training techniques, comprising vectors that produce bounding boxes corresponding to object positions in the input image. The channel and spatial contextualized sub-network further enhance the learning of the “box map”, enabling the more precise detection of small and intricately positioned objects.

This superior ability to detect small objects makes PF-FCN particularly effective for applications requiring the monitoring of rare or small targets. For example, in face detection, PF-FCN can reliably identify small facial features in crowded or low-light environments. In vehicle detection, it can accurately detect small or distant vehicles, enhancing real-time traffic monitoring and autonomous driving systems. In fire monitoring, PF-FCN’s precision in identifying small flames or smoke plumes can significantly improve early warning systems and response times to potential hazards. Overall, PF-FCN’s high $AP_{s}$ score and competitive FPS demonstrate its robustness and efficiency in detecting rare and small objects, making it a superior choice for various practical applications for which precision is paramount.

### 4.6. Ablation Study

This paper explores the impact of different parameter settings on detection performance. The experimental results cover changes in the K value, R-threshold, and threshold of the bounding box filter, as well as the contribution of GIoU and normalization improvements to performance, which are shown in Table 3. The bold data representation in the table means the best result in the same metrics.

**Performance overview.** The experimental results show that, when the K value increases from 7 to 26, $AP_{0.5∼0.95}$ and $AP_{0.5}$ are improved gradually. However, this improvement in accuracy accompanies a significant drop in FPS, indicating slower processing due to increased network complexity. At K values of 13, 20, and 26, the $AP_{0.5∼0.95}$ and $AP_{0.5}$ values almost remain the same, but FPS drops a little, indicating that increases of 13 provide the best balance between speed and accuracy.

The R-threshold value ranges from 0.1 to 0.9, whose step is 0.2. When the R-threshold is 0.1, the $AP_{0.5∼0.95}$ of this method is 53.2%, and the FPS is 34, indicating a relative balance between accuracy and speed. Gradually increasing the R-threshold to 0.5 results in a slight improvement in AP metrics, peaking at 53.4% of $AP_{0.5∼0.95}$ and maintaining a processing speed of 26 FPS. Beyond the R-threshold of 0.5, the accuracy starts to degrade, with an R-threshold of 0.9 dropping significantly to 51.3% of $AP_{0.5∼0.95}$. However, an FPS at R-threshold 0.9 increases to 32, indicating that a value of 0.5 provides the best balance between speed and accuracy.

In setting the threshold of the bounding box filter, a value of 0.7 provides high detection accuracy and a relatively fast processing speed. Although a value of 0.9 improves the processing speed, it leads to the discarding of some high-quality candidate-bounding boxes.

The engagement of GIoU and normalization both have a positive impact on the performance of the detector. The addition of GIoU slightly improves the performance of $AP_{0.5∼0.95}$. In the context of an ablation study, the notation “+GIoU” indicates the use of Generalized Intersection over Union (GIoU) as a replacement of the original Intersection over Union (IoU) metric. This replacement aims to improve the performance of object detection models. GIoU extends IoU by incorporating an additional penalty term that takes into account the distance between the predicted and ground truth boxes even when they do not overlap. This extension aims to address the limitations of IoU by providing a more informative and robust measure of bounding box regression.

**Analysis.** The K value, R-threshold, and threshold of the bounding box filter have a significant impact on detection performance. The increase in K value does improve accuracy, especially for large-sized object detection ($AP_{l}$), but the improvement for small objects ($AP_{s}$) is not obvious due to the difficulty of larger meshes in capturing small-sized details. Moderate K values, such as 10, 13, and 20, have less of an impact on FPS while maintaining high accuracy, which is the best choice for most practical applications.

The analysis of the impact of R-threshold values on detection-method performance illustrates the balance between accuracy and processing speed. An optimal R-threshold of 0.5 emerged as the most effective setting, improving accuracy for a variety of object sizes without significantly affecting speed. It suggests that a moderate threshold facilitates a refined selection process, effectively balancing reducing false positives and retaining true positive detections. However, a too-high or too-low R-threshold value can result in reduced accuracy due to overly conservative filtering or an increased processing speed at the expense of accuracy.

The threshold setting of the bounding box filter shows a balance between the threshold and the performance. Lower thresholds slightly reduce FPS while maintaining high accuracy. But higher thresholds significantly improve FPS at the expense of accuracy.

GIoU plays a role in improving bounding box regression accuracy. Normalization also helps improve performance because it improves adaptability with objects of different sizes and scales.

Overall, these experiments highlight the importance of the tuning network structure and post-processing steps in object detection tasks, as well as how detection performance can be optimized through appropriate improvements.

**Limitations.** Although PF-FCN exhibits significant advantages in terms of accuracy and speed, it also has several limitations. First, PF-FCN may face challenges when processing scenes with a high object density. Because PF-FCN does not localize a region of interest, overlapping or closely located objects may be missed or misclassified. Second, PF-FCN may struggle with objects of extreme sizes or uncommon aspect ratios because it does not have proposals with multiple scales and aspect ratios. Third, PF-FCN involves an inherent class imbalance between background classes and object classes because PF-FCN considers every position in its feature map as a potential object center.

## 5. Conclusions

In summary, the box map is the primary innovation of the proposed PF-FCN. This outstanding contribution has three distinct outputs, i.e., a bounding box-size map, a relevance-score map, and a classification-score map. Different from traditional proposal-based detectors that rely on a separate step to identify region proposals, PF-FCN directly associates image pixels with the bounding box coordinates and confidence scores of objects. The experimental results emphasize the effectiveness of the proposed free mechanism for object detection via MS COCO and PASCAL VOC data. In summary, PF-FCN represents significant progress in terms of balance accuracy and efficiency, and it has potential for real-time object-detection applications. A frontier research direction is to improve the detection accuracy and processing speed of proposal-free detectors in highly complex images and tiny object-detection scenarios. In the future, PF-FCN can be expanded to extract keypoints in 3D objects by leveraging its robust feature extraction and contextual modeling capabilities, potentially enhancing applications in 3D object recognition and reconstruction [53].

## Figures and Tables

**Table 1:** Comparisons of different methods on the MS COCO dataset (all timing data are based on a GeForce GTX Titan X).

Method	Backbone	${\mathrm{AP}}_{0.5∼0.95}$	${\mathrm{AP}}_{0.5}$	${\mathrm{AP}}_{s}$	${\mathrm{AP}}_{m}$	${\mathrm{AP}}_{l}$	FPS
Proposal-based method							
Faster RCNN [20]	ResNet-101	34.9	55.7	15.6	38.7	50.9	5
Mask R-CNN [30]	ResNeXt-101	39.8	62.3	22.1	43.2	51.2	5
R-FCN [13]	ResNet-101	31.5	53.2	14.3	35.5	44.2	∼3
Proposal-free method							
DeNet [46]	ResNet-101	33.8	53.4	12.3	36.1	50.8	
CoupleNet [47]	ResNet-101	34.4	54.8	13.4	38.1	50.8	
D-FCN [48]	AI-ResNet	37.5	58.0	19.4	40.1	52.5	
YOLOv2 [49]	DarkNet-19	21.6	44.0	5.0	22.4	35.5	81
SSD [18]	ResNet-101	31.2	50.4	10.2	34.5	49.8	
RetinaNet800 [34]	ResNet-101	39.1	59.1	21.8	42.7	50.2	12
CornerNet [21]	Hourglass-104	40.6	56.4	19.1	42.8	54.3	∼3
CenterNet [22]	Hourglass-104	44.9	62.4	25.6	47.4	57.4	∼3
FoveaBox [25]	ResNet-101	42.1	61.9	24.9	46.8	55.6	
YOLOv7 [29]	ELAN	51.2	69.7	35.2	56.0	66.7	**160**
YOLOv8 [50]	CSPDarknet53	52.9	69.8	35.7	**59.3**	**70.7**	
YOLOv9 [51]	CSP	53.0	70.2	36.2	58.5	69.3	
DETR [52]	Transformer	42.8	63.0	20.8	47.1	61.7	
FCOS [23]	ResNet-101	44.7	64.1	27.6	47.5	55.6	∼4
PF-FCN	ResNet-101	**53.6**	**71.7**	**37.6**	57.5	67.7	26

**Table 2:** Comparisons of different methods using the PSCAL VOC dataset (all timing data are based on a GeForce GTX Titan X).

Method	Training Data	Backbone	${\mathrm{AP}}_{0.5}$(%)	s/img	FPS
Proposal-based method					
Faster RCNN [20]	07 + 12 + COCO	ResNet-101	83.8	3.36	0.30
R-FCN [13]	07 + 12 + COCO	ResNet-101	82.0	0.17	5.88
Proposal-free method					
YOLOv1 [19]	07 + 12 + COCO	GoogleNet	63.4	0.02	45
SSD [18]	07 + 12 + COCO	ResNet-101	74.3	0.02	**46**
CornerNet [21]	07 + 12 + COCO	Hourglass-104	82.5	0.14	∼7
CenterNet [22]	07 + 12 + COCO	Hourglass-104	87.1	0.14	∼7
FCOS [23]	07 + 12 + COCO	ResNet-101	87.3	0.12	8.33
PF-FCN	07 + 12 + COCO	ResNet-101	**89.6**	0.04	25

**Table 3:** Ablation study of different methods using the MS COCO dataset (all timing data are based on a GeForce GTX Titan X).

Method	Value	${\mathrm{AP}}_{0.5∼0.95}$	${\mathrm{AP}}_{0.5}$	${\mathrm{AP}}_{s}$	${\mathrm{AP}}_{m}$	${\mathrm{AP}}_{l}$	FPS
	7	50.9	62.8	34.3	56.6	67.6	**32**
	10	51.2	69.3	37.6	57.4	67.7	27
K of box	**13**	**53.4**	**71.6**	**37.6**	**57.5**	**67.7**	26
	20	53.4	71.5	37.6	57.5	67.7	23
	26	53.3	71.5	37.5	57.5	67.3	16
	0.1	53.2	71.5	37.4	57.4	67.6	**34**
	0.3	53.3	71.6	37.5	57.5	67.7	24
R-threshold	**0.5**	**53.4**	**71.6**	**37.6**	**57.5**	**67.7**	26
	0.7	52.1	70.3	36.8	56.5	66.6	27
	0.9	51.3	68.1	34.5	55.7	65.2	32
	0.1	53.3	71.7	37.5	57.4	67.7	18
Threshold of	0.3	53.4	71.5	37.6	57.4	67.7	22
bounding-	0.5	53.4	71.5	37.6	57.4	67.7	24
box filter	**0.7**	**53.4**	**71.6**	**37.6**	**57.5**	**67.7**	26
	0.9	49.6	70.1	36.3	57.4	67.5	**29**
**Improvement**							
+GIoU		53.4	71.6	37.2	57.1	67.7	26
+Normalization		**53.6**	**71.7**	**37.6**	**57.5**	**67.7**	**26**

## Data Availability

Data is contained within the article.

## References

[1] Zhao Z.Q., Zheng P., Xu S.T., Wu X. (2019). Object detection with deep learning: A review. IEEE Trans. Neural Netw. Learn. Syst..

[2] Ghasemi Y., Jeong H., Choi S.H., Park K.B., Lee J.Y. (2022). Deep learning-based object detection in augmented reality: A systematic review. Comput. Ind..

[3] (2020). Deep Learning in Computer Vision: Principles and Applications.

[4] Khaleefah S.H., Mostafa S.A., Mustapha A., Nasrudin M.F. (2020). Review of local binary pattern operators in image feature extraction. Indones. J. Electr. Eng. Comput. Sci..

[5] Girshick R., Donahue J., Darrell T., Malik J. Rich feature hierarchies for accurate object detection and semantic segmentation. Proceedings of the IEEE Conference on Computer Vision and Pattern Recognition.

[6] He K., Zhang X., Ren S., Sun J. (2015). Spatial pyramid pooling in deep convolutional networks for visual recognition. IEEE Trans. Pattern Anal. Mach. Intell..

[7] Girshick R. Fast r-cnn. Proceedings of the IEEE International Conference on Computer Vision.

[8] Bachute M.R., Subhedar J.M. (2021). Autonomous driving architectures: Insights of machine learning and deep learning algorithms. Mach. Learn. Appl..

[9] Chen L., Lin S., Lu X., Cao D., Wu H., Guo C., Wang F.Y. (2021). Deep neural network based vehicle and pedestrian detection for autonomous driving: A survey. IEEE Trans. Intell. Transp. Syst..

[10] Iqbal M.J., Iqbal M.M., Ahmad I., Alassafi M.O., Alfakeeh A.S., Alhomoud A. (2021). Real-time surveillance using deep learning. Secur. Commun. Netw..

[11] Fang Q., Li H., Luo X., Ding L., Luo H., Rose T.M., An W. (2018). Detecting non-hardhat-use by a deep learning method from far-field surveillance videos. Autom. Constr..

[12] Jokanovic B., Amin M., Ahmad F. (2016). Radar fall motion detection using deep learning. Proceedings of the 2016 IEEE Radar Conference (RadarConf).

[13] An S., Pu X., Zhou S., Wu Y., Li G., Xing P., Hu C. (2022). Deep learning enabled neck motion detection using a triboelectric nanogenerator. ACS Nano.

[14] Su Z., Adam A., Nasrudin M.F., Ayob M., Punganan G. (2023). Skeletal Fracture Detection with Deep Learning: A Comprehensive Review. Diagnostics.

[15] Adam A., Rahman A.H.A., Sani N.S., Alyessari Z.A.A., Mamat N.J.Z., Hasan B. (2021). Epithelial layer estimation using curvatures and textural features for dysplastic tissue detection. CMC-Comput. Mater. Contin.

[16] Abbasi M., Shahraki A., Taherkordi A. (2021). Deep learning for network traffic monitoring and analysis (NTMA): A survey. Comput. Commun..

[17] Chen C., Liu B., Wan S., Qiao P., Pei Q. (2020). An edge traffic flow detection scheme based on deep learning in an intelligent transportation system. IEEE Trans. Intell. Transp. Syst..

[18] Liu W., Anguelov D., Erhan D., Szegedy C., Reed S., Fu C.Y., Berg A.C. (2016). Ssd: Single shot multibox detector. European Conference on Computer Vision.

[19] Redmon J., Divvala S., Girshick R., Farhadi A. You only look once: Unified, real-time object detection. Proceedings of the IEEE Conference on Computer Vision and Pattern Recognition.

[20] Ren S., He K., Girshick R., Sun J. (2016). Faster r-cnn: Towards real-time object detection with region proposal networks. IEEE Trans. Pattern Anal. Mach. Intell..

[21] Law H., Deng J. Cornernet: Detecting objects as paired keypoints. Proceedings of the European Conference on Computer Vision (ECCV).

[22] Duan K., Bai S., Xie L., Qi H., Huang Q., Tian Q. Centernet: Keypoint triplets for object detection. Proceedings of the IEEE/CVF International Conference on Computer Vision.

[23] Tian Z., Shen C., Chen H., He T. Fcos: Fully convolutional one-stage object detection. Proceedings of the IEEE/CVF International Conference on Computer Vision.

[24] Dong Z., Li G., Liao Y., Wang F., Ren P., Qian C. Centripetalnet: Pursuing high-quality keypoint pairs for object detection. Proceedings of the IEEE/CVF Conference on Computer Vision and Pattern Recognition.

[25] Kong T., Sun F., Liu H., Jiang Y., Li L., Shi J. (2020). Foveabox: Beyound anchor-based object detection. IEEE Trans. Image Process..

[26] Zand M., Etemad A., Greenspan M. (2022). Objectbox: From centers to boxes for anchor-free object detection. European Conference on Computer Vision.

[27] Tan M., Pang R., Le Q.V. Efficientdet: Scalable and efficient object detection. Proceedings of the IEEE/CVF Conference on Computer Vision and Pattern Recognition.

[28] Li C., Li L., Jiang H., Weng K., Geng Y., Li L., Wei X. (2022). YOLOv6: A single-stage object detection framework for industrial applications. arXiv.

[29] Wang C.Y., Bochkovskiy A., Liao H.Y.M. (2022). YOLOv7: Trainable bag-of-freebies sets new state-of-the-art for real-time object detectors. arXiv.

[30] He K., Gkioxari G., Dollár P., Girshick R. Mask r-cnn. Proceedings of the IEEE International Conference on Computer Vision.

[31] Cai Z., Vasconcelos N. (2019). Cascade R-CNN: High quality object detection and instance segmentation. IEEE Trans. Pattern Anal. Mach. Intell..

[32] Ding J., Niu S., Nie Z., Zhu W. (2024). Research on Human Posture Estimation Algorithm Based on YOLO-Pose. Sensors.

[33] Peng J., Ouyang C., Peng H., Hu W., Wang Y., Jiang P. (2024). MultiFuseYOLO: Redefining Wine Grape Variety Recognition through Multisource Information Fusion. Sensors.

[34] Lin T.Y., Goyal P., Girshick R., He K., Dollár P. Focal loss for dense object detection. Proceedings of the IEEE International Conference on Computer Vision.

[35] Guo M.H., Xu T.X., Liu J.J., Liu Z.N., Jiang P.T., Mu T.J., Hu S.M. (2022). Attention mechanisms in computer vision: A survey. Comput. Vis. Media.

[36] Mo Y., Wu Y., Yang X., Liu F., Liao Y. (2022). Review the state-of-the-art technologies of semantic segmentation based on deep learning. Neurocomputing.

[37] Sahoo P.K., Panda M.K., Panigrahi U., Panda G., Jain P., Islam M.S., Islam M.T. (2024). An Improved VGG-19 Network Induced Enhanced Feature Pooling For Precise Moving Object Detection In Complex Video Scenes. IEEE Access.

[38] Lin T.Y., Dollár P., Girshick R., He K., Hariharan B., Belongie S. Feature pyramid networks for object detection. Proceedings of the IEEE Conference on Computer Vision and Pattern Recognition.

[39] Saif A.F.M.S., Mahayuddin Z.R. (2022). Vision based 3D Object Detection using Deep Learning: Methods with Challenges and Applications towards Future Directions. Int. J. Adv. Comput. Sci. Appl..

[40] Zulkifley M.A., Abdani S.R., Zulkifley N.H. (2019). Pterygium-Net: A deep learning approach to pterygium detection and localization. Multimed. Tools Appl..

[41] Karim T., Mahayuddin Z.R., Hasan M.K. (2023). Singular and Multimodal Techniques of 3D Object Detection: Constraints, Advancements and Research Direction. Appl. Sci..

[42] Nafea M.M., Tan S.Y., Jubair M.A., Abd Mustafa T. (2022). A Review of Lightweight Object Detection Algorithms for Mobile Augmented Reality. Int. J. Adv. Comput. Sci. Appl..

[43] Liu Y., Kang K.D. (2024). Filtering Empty Video Frames for Efficient Real-Time Object Detection. Sensors.

[44] Lin T.Y., Maire M., Belongie S., Hays J., Perona P., Ramanan D., Zitnick C.L. (2014). Microsoft coco: Common objects in context. Computer Vision–ECCV 2014: 13th European Conference, Zurich, Switzerland, September 6–12, 2014, Proceedings, Part V 13.

[45] Everingham M., Eslami S.A., Van Gool L., Williams C.K., Winn J., Zisserman A. (2015). The pascal visual object classes challenge: A retrospective. Int. J. Comput. Vis..

[46] Tychsen-Smith L., Petersson L. Denet: Scalable real-time object detection with directed sparse sampling. Proceedings of the IEEE International Conference on Computer Vision.

[47] Zhu Y., Zhao C., Wang J., Zhao X., Wu Y., Lu H. Couplenet: Coupling global structure with local parts for object detection. Proceedings of the IEEE International Conference on Computer Vision.

[48] Dai J., Qi H., Xiong Y., Li Y., Zhang G., Hu H., Wei Y. Deformable convolutional networks. Proceedings of the IEEE International Conference on Computer Vision.

[49] Redmon J., Farhadi A. YOLO9000: Better, faster, stronger. Proceedings of the IEEE Conference on Computer Vision and Pattern Recognition.

[50] Jocher G. YOLOv8 Release v8.1.0.; 2024. 3, 7. https://github.com/ultralytics/ultralytics/releases/tag/v8.1.0.

[51] Wang C.Y., Yeh I.H., Liao H.Y.M. (2024). YOLOv9: Learning What You Want to Learn Using Programmable Gradient Information. arXiv.

[52] Dai Z., Cai B., Lin Y., Chen J. Up-detr: Unsupervised pre-training for object detection with transformers. Proceedings of the IEEE/CVF Conference on Computer Vision and Pattern Recognition.

[53] Wu Y., Chen X., Huang X., Song K., Zhang D. (2024). Unsupervised distribution-aware keypoints generation from 3D point clouds. Neural Netw..

